# Immunology of Lynch Syndrome

**DOI:** 10.1007/s11912-021-01085-z

**Published:** 2021-06-14

**Authors:** Danielle M. Pastor, Jeffrey Schlom

**Affiliations:** 1 Laboratory of Tumor Immunology and Biology, Center for Cancer Research, National Cancer Institute, National Institutes of Health, Bethesda, MD USA; 2 NIH Hematology Oncology Fellowship Program, National Institutes of Health, Bethesda, MD USA

**Keywords:** Lynch syndrome, Colorectal cancer, Microsatellite instability (MSI), Vaccines, Immunotherapy, Neoantigens

## Abstract

**Purpose of Review:**

Patients with Lynch syndrome have a high probability of developing colorectal and other carcinomas. This review provides a comprehensive assessment of the immunologic aspects of Lynch syndrome pathogenesis and provides an overview of potential immune interventions for patients with Lynch syndrome polyps and Lynch syndrome–associated carcinomas.

**Recent Findings:**

Immunogenic properties of the majority of Lynch syndrome polyps and associated cancers include microsatellite instability leading to a high mutational burden and the development of novel frameshift peptides, i.e., neoantigens. In addition, patients with Lynch syndrome develop T cell responses in the periphery and in the tumor microenvironment (TME) to tumor-associated antigens, and a proinflammatory cytokine TME has also been identified. However, Lynch syndrome lesions also possess immunosuppressive entities such as alterations in MHC class I antigen presentation, TGFβ receptor mutations, regulatory T cells, and upregulation of PD-L1 on tumor-associated lymphocytes.

**Summary:**

The rich immune microenvironment of Lynch syndrome polyps and associated carcinomas provides an opportunity to employ the spectrum of immune-mediating agents now available to induce and enhance host immune responses and/or to also reduce immunosuppressive entities. These agents can be employed in the so-called prevention trials for the treatment of patients with Lynch syndrome polyps and for trials in patients with Lynch syndrome–associated cancers.

## Introduction

The concept of immunotherapy as an anti-cancer strategy has exploded across the oncologic stage over recent years and commanded the attention of those charged with the mission of caring for patients with malignancies. Numerous host-related variables, factors related to tumor microenvironment (TME), and tumor autonomous mechanisms have been postulated as predictive biomarkers for treatment response in this setting. Tumor mutational load and extent of microsatellite instability (MSI) are recognized as strong determinants of checkpoint inhibitor–based immunotherapy efficacy; likewise, microsatellite stability (MSS) and the proficiency of the associated process of mismatch repair (MMR) have become increasingly influential factors in the determination of the type of treatment to be used to combat malignancy.

The majority of colon and rectal adenocarcinomas are MSS and proficient with respect to MMR. Approximately 12–15% of colorectal cancers (CRCs), however, exhibit MSI and are considered mismatch repair deficient (dMMR), characteristics suggested to be largely responsible for their immunogenic phenotype, in contrast to the features inherent to their immunologically inert MSS counterparts. Consequently, those patients with gastrointestinal cancers that exhibit MSI have shown greater responses to checkpoint inhibition immunotherapy than patients bearing MSS tumors [1–4].

Lynch syndrome (LS) is the most common cause of hereditary colon cancer, with a prevalence noted to be as high as 1 in 279 individuals in the general population and as common as 1 in 35 patients with colon cancer [5, 6]. The overwhelming majority of colon cancers arising in this setting exhibit MSI and develop proximal to the splenic flexure [7–10]. Synchronous and metachronous tumors are characteristic of this syndrome, with individuals typically in their mid-40s when they first develop CRC [7, 11, 12]; disease phenotype is highly variable and heavily influenced by the mutation present, as the syndrome is a disorder of molecular genetic heterogeneity. Individuals with germline mutations in disease-defining DNA MMR genes are not only at risk to develop colorectal cancer at relatively early ages, but also carry higher risks of developing extracolonic cancers, such as endometrial, gastric, ovarian, biliary, and urinary tract malignancies [13, 14]. Small intestinal and brain tumors have also been ascribed to the syndrome, although these cancers are substantially less prevalent than others [13]. Table 1 delineates the key features of Lynch syndrome. Unlike the 10–15 years necessary for the transformation of adenoma to carcinoma in individuals of average risk, malignant transformation in LS occurs at accelerated rates, usually within 1–3 years, underscoring the necessity for proper counseling and intense surveillance [15, 16]. Lynch syndrome–associated colorectal cancers (LS-CRCs) display distinct histopathological features; they are commonly diploid, poorly differentiated, and characterized by a heavy infiltration of lymphocytes, medullary growth pattern, mucinous, or signet ring cell differentiation, with a “Crohn’s-like reaction” [13, 15, 17–19].

**Table 1:** Features of Lynch syndrome

• Increased risk for development of colonic and extracolonic malignancies	
• Early age of onset of first malignancy (typically colon or endometrial)	
• Germline mutations of MMR genes	
• Autosomal dominant inheritance pattern	
• Cancers exhibiting microsatellite instability	
• Proximal location of colon cancers	
• Development of extracolonic cancers	
• Accelerated rate from adenoma to carcinoma	
• Poorly differentiated tumors	
• Increased density of tumor-infiltrating lymphocytes and heightened T cell responses	

## Surveillance and Lynch Syndrome

A preventive approach is key to the overall management of LS, with patient compliance with surveillance colonoscopy critical for successful risk reduction. LS-CRC is typically early stage at diagnosis, often without nodal involvement. Thus, LS-CRC often represents resectable disease, with total colectomy with ileorectal anastomosis considered primary treatment for patients with cancer or with precursor lesion(s) not amenable to endoscopic resection. Prophylactic colectomy remains a controversial alternative and should be reserved only for those individuals for whom colonoscopies are difficult and pose risk greater than benefit and in circumstances in which compliance is anticipated to be challenging.

Lynch syndrome confers an increased lifelong risk to develop CRC that is not inconsequential. An integral facet in the management of LS is surveillance endoscopy with the objectives of identifying and removing precursor lesions prior to their progression to carcinoma. The development of cancer in this setting is attributed to the acceleration of this progression, rather than the presence of an increased number of precursor lesions with transformative potential. Thus, interval surveillance via regularly scheduled colonoscopies is imperative to risk reduction and is recommended by the American Gastroenterological Association to be performed every 1–2 years starting at 20–25 years of age (or 5 years before the youngest age of diagnosis of colorectal cancer in an affected family member if diagnosis occurs earlier) [20]. Despite clearly established evidence regarding the association between surveillance colonoscopy and decreased LS-CRC burden and decreased LS-CRC–associated mortality, compliance remains suboptimal for many individuals [21].

## Microsatellite Instability in Lynch Syndrome

As one of the most common hereditary cancer syndromes, LS gives rise to 3–5% of MSI-CRCs. Hallmarks of tumors that develop in this setting are MSI and dMMR, not only in the CRCs arising in patients with this inheritable disease, but also in the extracolonic malignancies known to occur at increased rates in individuals affected by this susceptibility syndrome. While LS-CRC represents the smaller constituent of MSI-CRCs, the remaining 10–12% of MSI-CRC tumors are comprised by those developing in a sporadic pattern, in individuals lacking any notable familial predisposition.

Lynch syndrome is inherited through an autosomal dominant pattern [11, 22]. The cumulative lifetime risk of developing CRC in this patient population can be as high as 60–80% without surveillance, although substantially lower penetrance has been reported, depending on the gene involved [23]. It should be noted that while the terms LS and hereditary non-polyposis colon cancer (HNPCC) are often used interchangeably, LS specifically refers to patients and family members with known pathognomonic germline mutations, and HNPCC refers to individuals and families who meet the Amsterdam criteria [24, 25]. In this regard, the designation of HNPCC also encompasses cases of familial CRC that are MSI and exhibit features of LS but lack germline mutations involving mismatch repair genes (“Lynch-like” syndromes) or appear to be familial but are MSS and lack germline mutations (“familial colorectal cancer type X”) [24, 26–30].

**Fig. 1:**
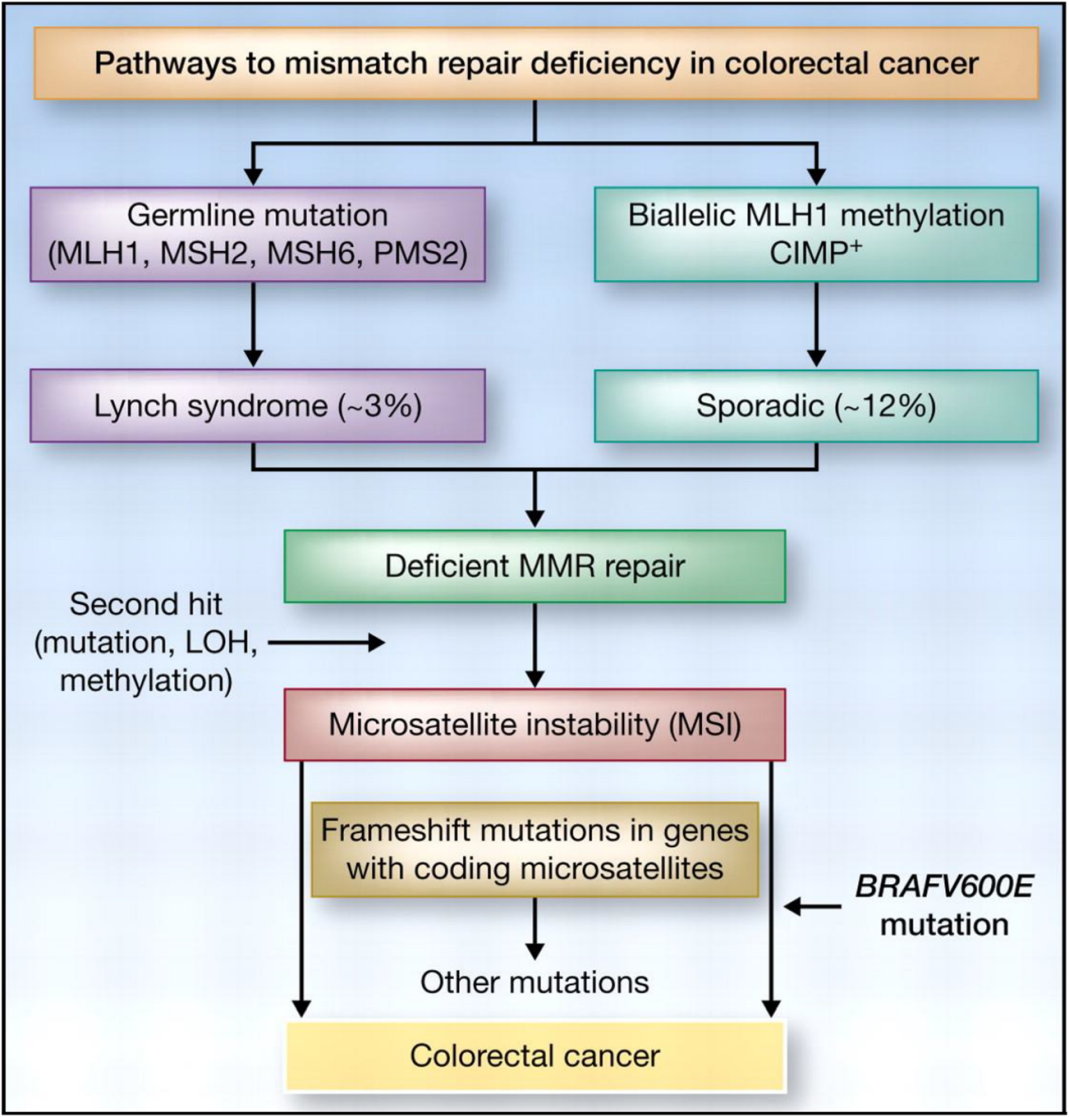
Two molecular pathways can lead to colorectal cancers with microsatellite instability. These include germline mutations in a mismatch repair (MMR) gene followed by a second hit or the more common, nonfamilial form of MSI, which is due to epigenetic inactivation of MLH1 occurring in a background of hypermethylation. Figure from ref. [35]

Pathogenic germline mutations in the DNA MMR genes mutL homolog 1 (MLH1), mutS homolog 2 (MSH2), mutS homolog 6 (MSH6), and post-meiotic segregation increased 2 (PMS2) are defining features of LS [31]. Although the inheritance pattern is autosomal dominant, pathology ultimately results from a second event involving the unaffected allele. Germline deletions have also been recognized as LS-associated mutations [32]. A third alternative etiology that has been proposed in recent years is germline epimutations [33, 34]. Figure 1 illustrates the two major pathways for MSI in colorectal malignancies.

## Immunogenicity of Lynch Syndrome

**Fig. 2:**
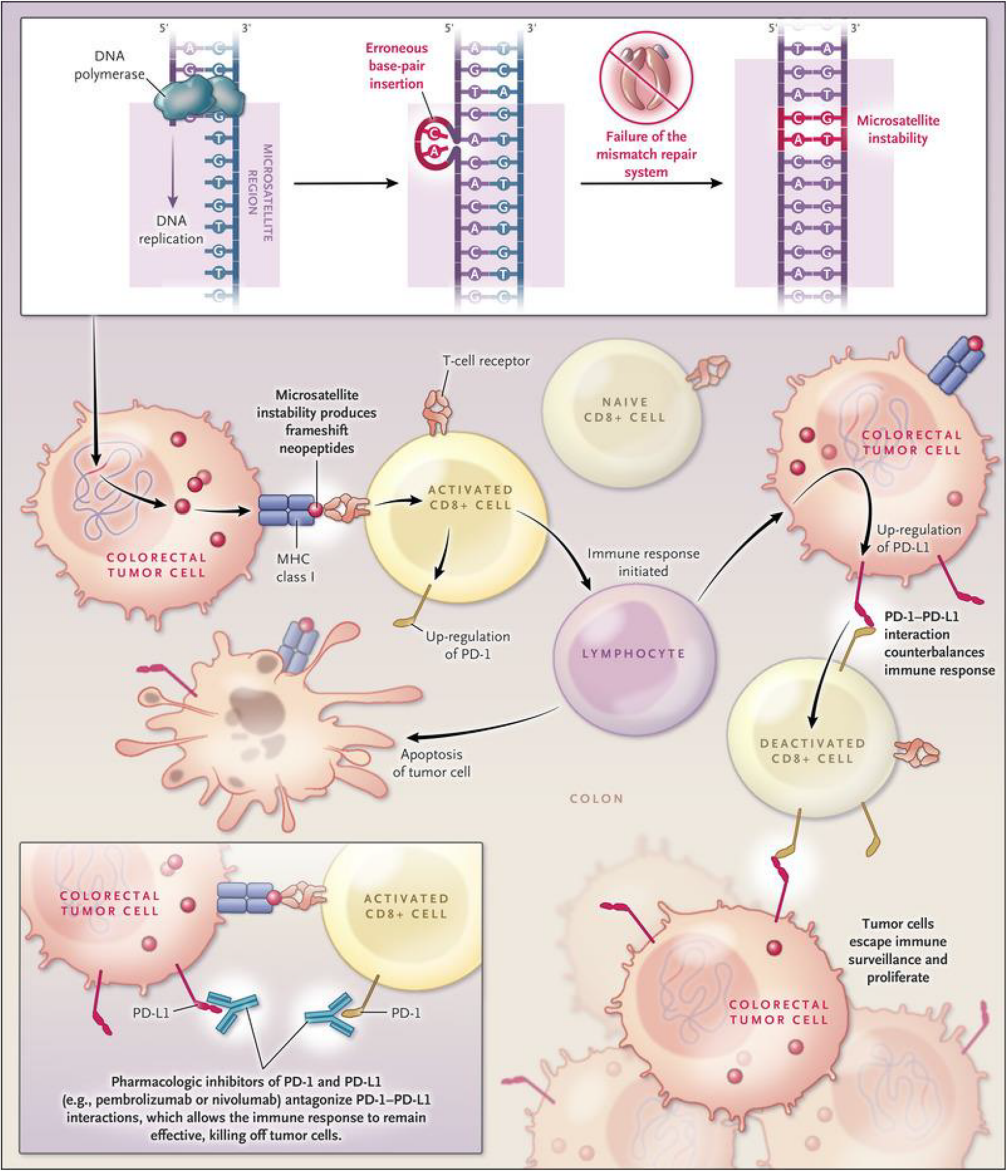
Targeting of colorectal cancers in patients with deficient mismatch repair with the use of immune checkpoint inhibitors. Deficient DNA mismatch repair in tumors results in microsatellite instability and thus extensive insertions and deletions in coding regions that lead to frameshift mutations. These mutations may generate neoantigens and induce a host immune response to the tumor that can potentially be enhanced by immune checkpoint blockade. Figure from ref. [45]

The prevailing theory for the immunogenicity of LS is rooted in its inherent “mutator phenotype.” Disease-defining germline mutations of mismatch repair genes result in the accumulation of frameshift mutations within coding sequences, ultimately leading to the synthesis of abundant “neoantigens” — the principal provocateurs of immunoreactivity in this disorder that can potentially behave as tumor-specific antigens (Table 2) [36–40]. Frameshift mutations can be beneficial or detrimental to immunosurveillance in LS carcinogenesis. In some cases, dMMR-related neoantigens may potentially be recognized and cleared by the immune system; in others, frameshift mutations may potentially facilitate immune escape via the alteration of cell surface proteins responsible for antigen processing and presentation [26, 41, 42]. CD8^+^ T cells appear to play a role in the recognition of these immunogenic neopeptides; in fact, CD8^+^ T cell infiltrates are present in LS-CRC, notably and abundantly in cancer nests, with infiltration suggested to be associated with improved prognoses in LS [26, 43, 44]. Figure 2 depicts the interplay between tumor-infiltrating lymphocytes (TILs) and colorectal cancer cells. This complex relationship between tumor and constituents of the TME is especially critical in LS-CRCs, as it plays a principal role in the balance between tumor elimination and development. Frameshift peptide (FSP)–specific immune responses are prominent in TILs in LS-CRC, with FSPs inducing robust interferon production from TILs [44]. FSP-specific TILs from LS-CRCs also retain their cytotoxic potential, demonstrated by the lysis of cancer cells in vitro [44]. High levels of TILs are not only exhibited by CRCs in LS but also apparent in extracolonic tumors from LS patients.

**Table 2:** Immunogenic properties of Lynch syndrome–associated colorectal cancers

• Deficient DNA mismatch repair	
• Microsatellite instability	
• High mutational burden in genes encompassing coding microsatellites	
• Mutation-induced novel frameshift peptides (creation of “neoantigens”)	
• Proinflammatory cytokine tumor microenvironment	
• Peritumoral lymphoid nodules	
• Dense T cell lymphocyte tumor infiltration with heightened T cell response	
• Upregulation of caspase 3 and natural killer cell inhibitory ligand (KIR2DL1)

## Antibody Responses in Lynch Syndrome Patients

Antibody responses against FSPs are not only detectable in patients with LS-CRC, but also endogenously induced in individuals with known germline mutations without a history of tumor development, although the response produced is of a lesser degree in the latter [40, 43]. Among those who have undergone colectomies for LS-CRC, humoral responses have been shown to be more robust in patients with the shortest intervals between resections and sera collection. The production of FSP-induced antibodies has also shown to be associated with higher stages of disease, i.e., in more patients with regional lymph node involvement than those with local disease [40]. These findings lay the groundwork for further study of a possible role for FSP-specific humoral responses as potential biomarkers in diagnostic applications; these responses may exist as potential adjuncts in the surveillance of patients with LS, as well as predictive markers with which to assess disease state and/or severity.

## T Cell Responses in Lynch Syndrome Patients

Frameshift peptide-specific effector T cell responses are detectable in the peripheral blood of both healthy LS patients without history of cancer and those with LS-CRC [44, 46–48]. In the evaluation of immune responses in these individuals, it was noted that the observed T cell responses were directed at 14 different FSP antigens with varying mutation frequencies predicted from human genome databases; it was discovered that the neoantigens derived from genes with high mutation frequencies that exhibit in vitro immunogenicity were common to both LS-CRC and sporadic MSI-CRC, demonstrating a “shared landscape” [44, 49]. Among these genes were HT001, AIM2, TAF1B, TGFβRII, and FSP06 [46, 50–55]. Of interest, over half of LS adenomas exhibit TGFβRII mutations; the finding that TGFβRII mutations are expressed in a substantial fraction of early formed lesions in LS lends support to the concept that immunoediting plays a role in both the early and late phases in the progression of adenoma to carcinoma in an effort to maintain immunologic homeostasis [56]. The fact that TGFβRII mutations are so prevalent in Lynch syndrome adenomas brings into play the dual and multifunctional roles of TGFβ in tumorigenesis. While TGFβ has been described to play an anti-neoplastic role in early transformation processes [57, 58], it has also been shown to play a pro-neoplastic role in many cancer types. The increased activity of regulatory T cells (Tregs) and driving epithelial tumor cells to the more mesenchymal phenotype, rendering them more resistant to therapy, has been shown to be mediated by TGFβ [59–61].

**Fig. 3:**
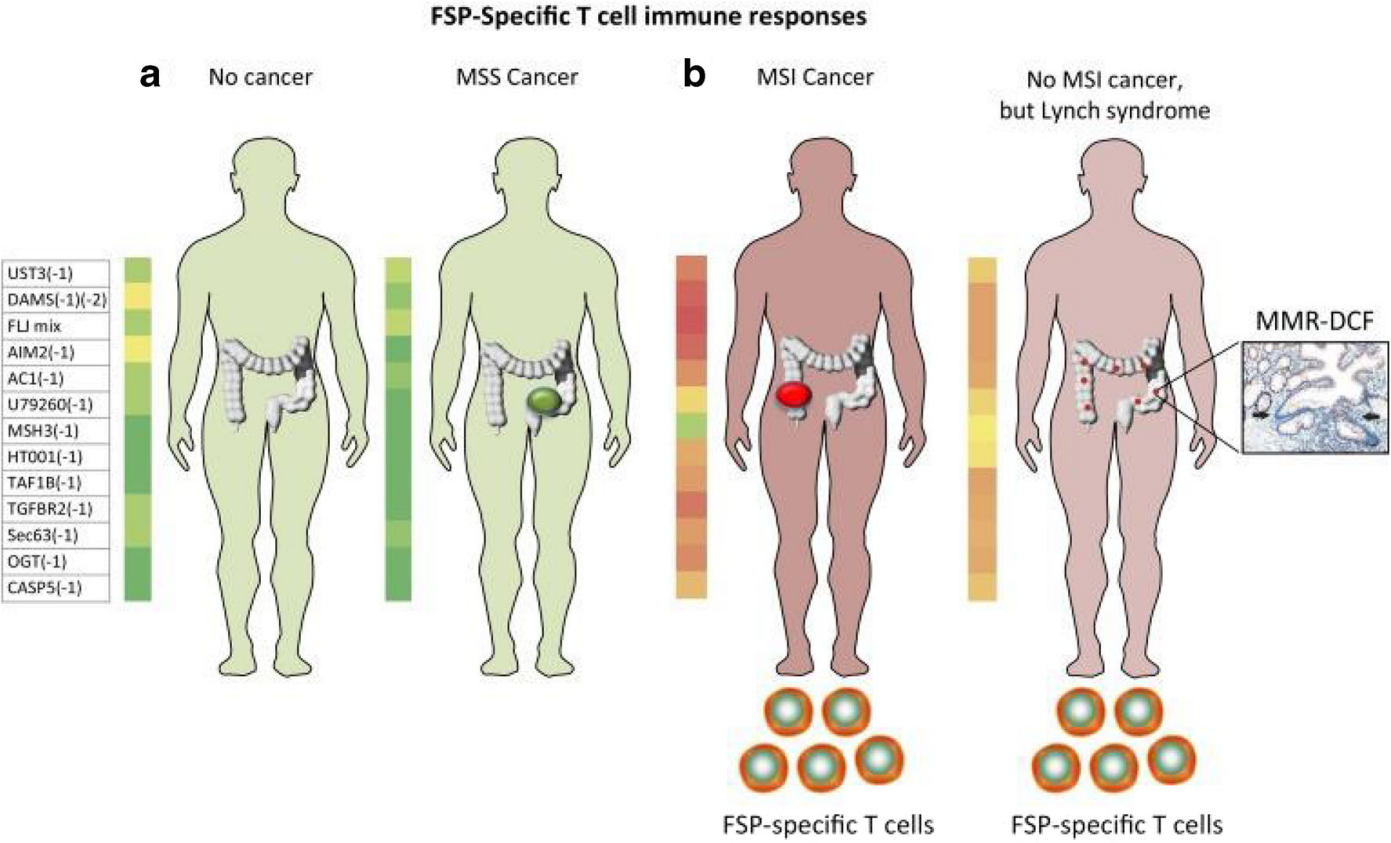
Frameshift peptide (FSP)–specific T cells can be generated in patients with microsatellite-unstable (MSI) cancer and LS mutation carriers. The prevalence of T cell responses against defined FSP antigens is lower or absent in healthy individuals or patients with microsatellite-stable (MSS) cancer (**a**) vs patients with MSI cancer and LS mutation carriers (**b**). The heat maps show the increasing frequency of FSP from green to red. Figure from ref. [37]

Frameshift peptides may be generated as early as haploinsufficiency if one MMR gene becomes relevant, which may explain the activated immune response against neopeptides in healthy LS mutation carriers without history of tumor development (Fig. 3) [43, 44]. The phenomenon of detectable immune response in healthy individuals known to carry LS-associated germline mutations may be partly explained by the “elimination phase” theory: the concept that immunoediting occurs early and consistently in this disorder, enabling successful elimination of many lesions at early stages secondary to efficient immune surveillance [37, 62, 63]. This ongoing process of recognition and elimination may be a contributing factor to the limited penetrance that occurs with certain mutations in LS [64]. It is the disruption of the ongoing processes of tumor recognition and elimination when tumor escape becomes prevalent; this is when further immune intervention is required, as will be discussed below.

## Immune Surveillance and Influence in Tumor Development in Lynch Syndrome

**Fig. 4:**
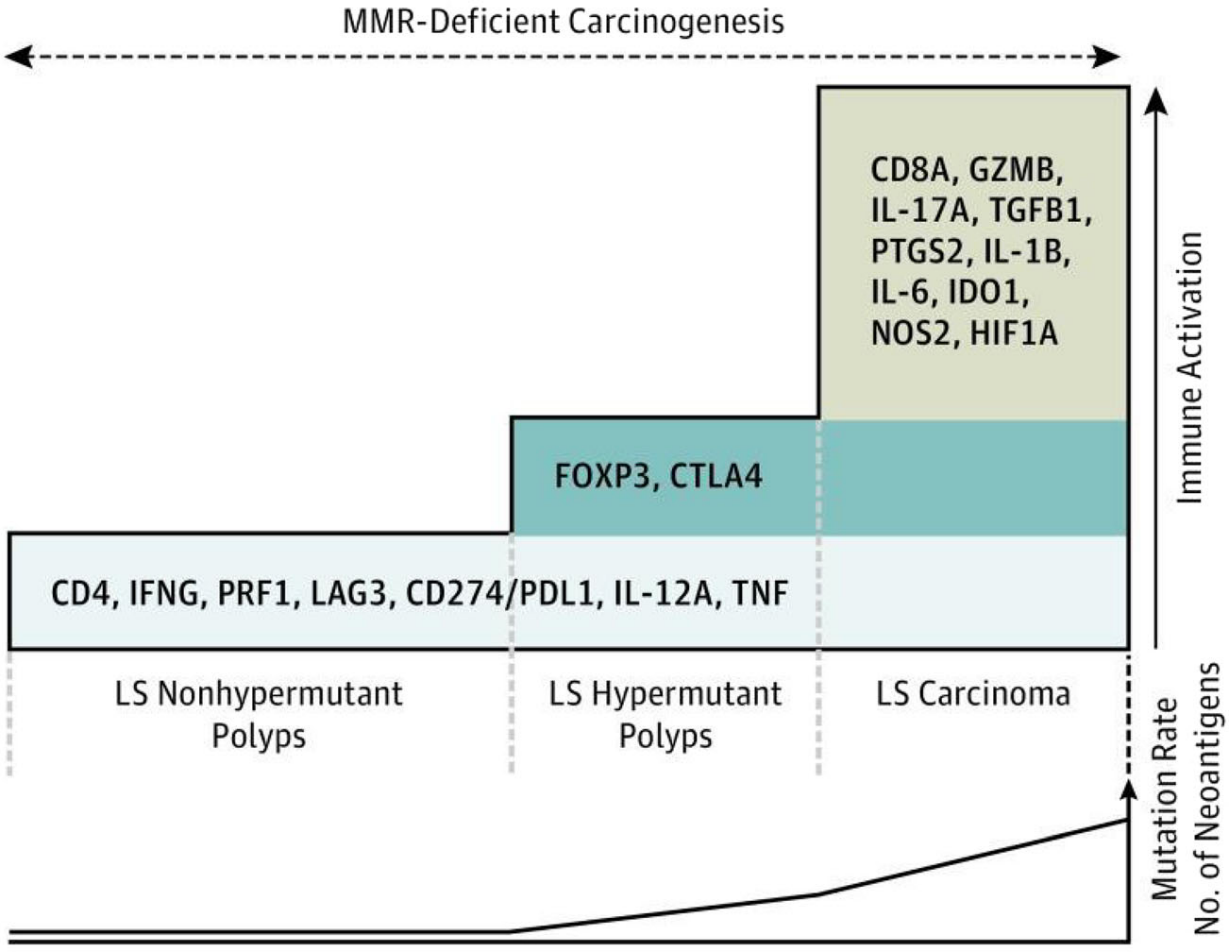
Schematic model of the immune activation in Lynch syndrome carcinogenesis. LS polyps display immune activation characterized by CD4 T cells, proinflammatory, and checkpoint molecules. Progression of mutational rate and evolution into carcinomas activate additional immune pathways. This may eventually lead to the development of immune tolerance and thus evasion. Figure from ref. [66]

The incorporation of immunotherapy into cancer treatment regimens and the use of scoring systems utilizing immunologic markers in the prognoses of malignant disease underscore the integral role that the immune system plays in the processes of carcinogenesis [1–4, 65]. The pronounced cytotoxic T cell activity in LS renders the disease an excellent model to evaluate the processes of immune surveillance and evasion. Premalignant LS lesions arise in a robust immune microenvironment, within which T cell infiltration is a predominant process [66, 67]. LS adenomas are not only characterized by higher numbers of T lymphocytes than sporadic polyps, but they also exhibit higher mRNA expression levels of CD4, IFNγ, LAG3, CD274/PDL1, IL12A, and TNFα compared with non-LS polyps (Fig. 4) [66, 67]. Strikingly, the upregulation of immune-related genes in these precursor lesions was shown to be independent of mutational rate and neoantigen load, suggesting that immune activation occurs early in the process of LS-associated tumorigenesis and is not merely the consequence of accumulating somatic mutations, thereby supporting the concept of immunoprevention as a prophylactic strategy in the management of LS [66]. LAG3 (or cluster of differentiation 223 [CD223]) was the most significantly upregulated gene of the subset evaluated above; of interest, the LAG3 protein is an immune checkpoint receptor that, like PD-1 and CTLA-4, negatively regulates the proliferation, activation, and homeostasis of T cells and also plays a role in Treg suppressive function [68–70]. Multiple studies are currently evaluating anti-LAG3 treatment in advanced cancers, as monotherapy or in combination with other checkpoint inhibitors (NCT03005782, NCT01968109, NCT03044613, NCT03607890, NCT03642067).

## Immunosuppressive Entities in Lynch Syndrome

Frameshift mutations occur at all stages of tumor development in LS with some genes acquiring higher frequencies of mutations early on in the process and others activated later on (Table 3). Mutations in the genes BAX2 and TGFβRII occur early and are found in over half of LS adenomas; in addition, these genes continue to accumulate mutations during the progression towards high-grade adenomas [56, 71]. Mutations in other genes, such as PTHL3, feature more prominently in the later stages of adenoma to carcinoma transition [71]. These mutations occur in LS carcinomas at higher frequencies than in precursor lesions and include those found in genes associated with proinflammatory and metabolism pathways [66].

**Table 3:** Potential immunosuppressive entities in Lynch syndrome–associated colorectal cancers

• Alterations of HLA class I antigen presentation	
Truncating beta2-macroglobulin (*B2M*) mutations	
Transporter protein (*TAP1, TAP2*) mutations	
• Loss or downregulation of HLA class I heavy chains	
• Loss of HLA class II antigen presentation	
*RFX5* mutations	
*CIITA* mutations	
• Transforming growth factor beta type II receptor (*TGFβRII*) mutations	
• Presence of FOXP3^+^ regulatory T cells	
• Upregulated PD-L1 expression in tumor-infiltrating lymphocytes	

Type and intensity of immune response may reflect disease stage in Lynch syndrome. Early-stage tumors have been shown to be more densely infiltrated by activated CD8^+^ T cells than in advanced stages [72]; this high degree of tumor infiltration by activated CD8^+^ cells correlates with aberrant HLA class 1 expression [72]. Conversely, a lower density of immune cell infiltration has been observed in more advanced lymph node-positive tumors [72]. Immune response may also predict outcome, as high density of TILs correlates with improved outcome in LS-CRC patients [73]. Immune response may not only be a predictive factor in this sense, but also a means to intervene in the development of LS-CRC.

## Immune Evasion in Lynch Syndrome

**Fig. 5:**
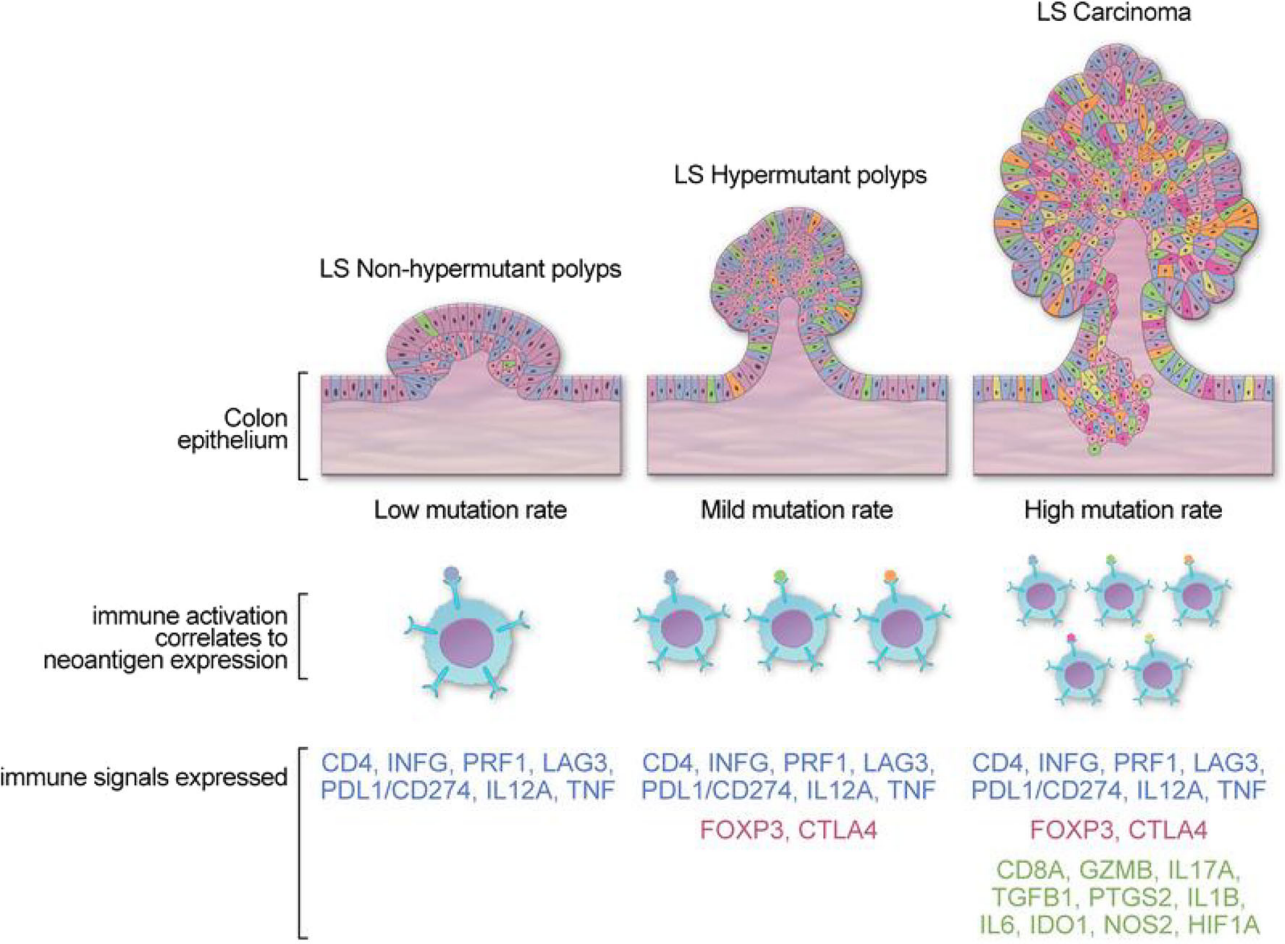
Correlation of immune activation neoantigen burden and adenoma to carcinoma formation in Lynch syndrome–related colorectal cancer. The different colors in the top panel represent intralesional mutation and/or neoantigen diversity. As the lesions progress to advanced adenomas, there is a rise in mutation/neoantigen burden and markers of immune tolerance. Figure from ref. [74]

Although the immune signature in premalignant lesions in LS described above by Chang et al. was determined to be independent of mutational rate and neoantigen load, the upregulation of the regulatory T cell–related gene FOXP3 and corresponding FOXP3^+^ T cell infiltration was specifically observed in dMMR hypermutated LS polyps (Fig. 5) [66]. Infiltration with FOXP3^+^ T cells has similarly been shown in mucosa adjacent to LS-CRCs, with lower density significantly correlating with the presence of β2M mutation [75]. Conversely, no such correlation was evident between FOXP3^+^ T cell infiltration in intratumoral or tumor-distant normal mucosa and β2M mutation status. The combination of impaired HLA class I antigen expression resulting from β2M mutation and the reduction of an immunosuppressive T cell population may heavily promote the proliferation of tumor cells that are less immunogenic and more inclined to evade surveillance in the process of immunoediting [75]. Rarely observed in MSS and sporadic MSI-CRCs, β2M mutations are present in approximately 30–40% of LS-CRCs [76–78]. Although identified as a principal culprit driving immune evasion in these CRCs, β2M mutations are, perhaps counterintuitively, associated with favorable outcomes, such as reduced metastases and relapse, as they are protected against the formation of distant organ metastases in patients with MMR-deficient CRCs, particularly in LS [42, 77, 79–81].

Beta-2-microglobulin (β2M) loss is rarely found in distant metastases or dMMR adenomas and has been associated with improved prognosis, particularly adding prognostic value to the increased CD8^+^ T cell levels found in LS-CRC [77, 82, 83]. A possible explanation for the favorable prognosis associated with β2M negative tumors is the concurrent activation of natural killer cell–mediated apoptosis, which is supported by the upregulation of caspase 3 and natural killer cell inhibitory ligand (KIR2DL1) seen in LS-CRC [77].

The upregulation of programmed death-ligand 1 (PD-L1) is suspected to contribute to immune evasion in LS-CRC. In LS-CRC, PD-L1 expression is more frequently observed on immune cells at the invasive margin; peri- and intratumoral macrophages also show extensive PD-L1 expression, while expression is rarely observed on tumor cells themselves [76]. LS tumors have lower PD-L1 expression than their sporadic MSI-H CRC counterparts, with expression predominantly demonstrated in a focal pattern and widely varied regarding whether expressed in both tumor cells and stromal macrophages or in only one type of cell versus the other [84, 85]. PD-L1 expression in TILs, but not tumor cells, has been shown to predict prognosis. Similarly, a significant association between high PD-L1 levels and high levels of CD3^+^ and CD8^+^ cells in LS has been recognized [76]. In this regard, germline mutational status (e.g., MLH1-/PMS2- vs MSH2-/MSH6-) may occupy a less critical role with respect to PD-L1 expression [84].

A complex interplay thus exists between PD-L1 expression and β2M loss in LS [76]. Both the downregulation of β2M and the upregulation of PD-L1 inhibitory signals can shield tumor cells from cytotoxic T cell–mediated apoptosis [76]. The upregulation of PD-L1 in TILs may indicate potential response to checkpoint inhibition, but the evident loss of β2M may ultimately result in decreased sensitivity to these therapies because of loss of MHC class I presentation [76].

## Modulation of Immune-Associated Genes in Lynch Syndrome

The modulation of immune-associated genes is undoubtedly a prominent mechanism contributing to the immune-evasive phenotype associated with Lynch syndrome. Examples of involved pathways with the upregulated genes in LS are those associated with antigen processing and presentation, apoptosis, natural killer cell–mediated cytotoxicity, inhibitory genes, and T cell activation (Table 4)*.* In contrast, genes that are known to influence antigen presentation, folding, assembling, and loading of MHC class I receptors that are downregulated in LS-CRC include *HLA-F*, *UBE2D2*, *SEC31A*, and *ITGB5* [76].

**Table 4:** Immune-related genes upregulated in Lynch syndrome–associated colorectal cancers

• Antigen processing and presentation	
*CIITA*, *CD74*, MHC class II receptors	
• Apoptosis	
*CASP3*, *FAS*, *BCL2*, *TRAILR2*	
• Natural killer cell–mediated cytotoxicity	
*CD226, GZMA, GZMH, CXCL16*	
• Inhibitory genes	
*IDH1*, *KIR2DL1*, *IL18BP*	
• T cell activation	
*NFATC1*, *CD86*, *CD247*, *MALT1*	

**Fig. 6:**
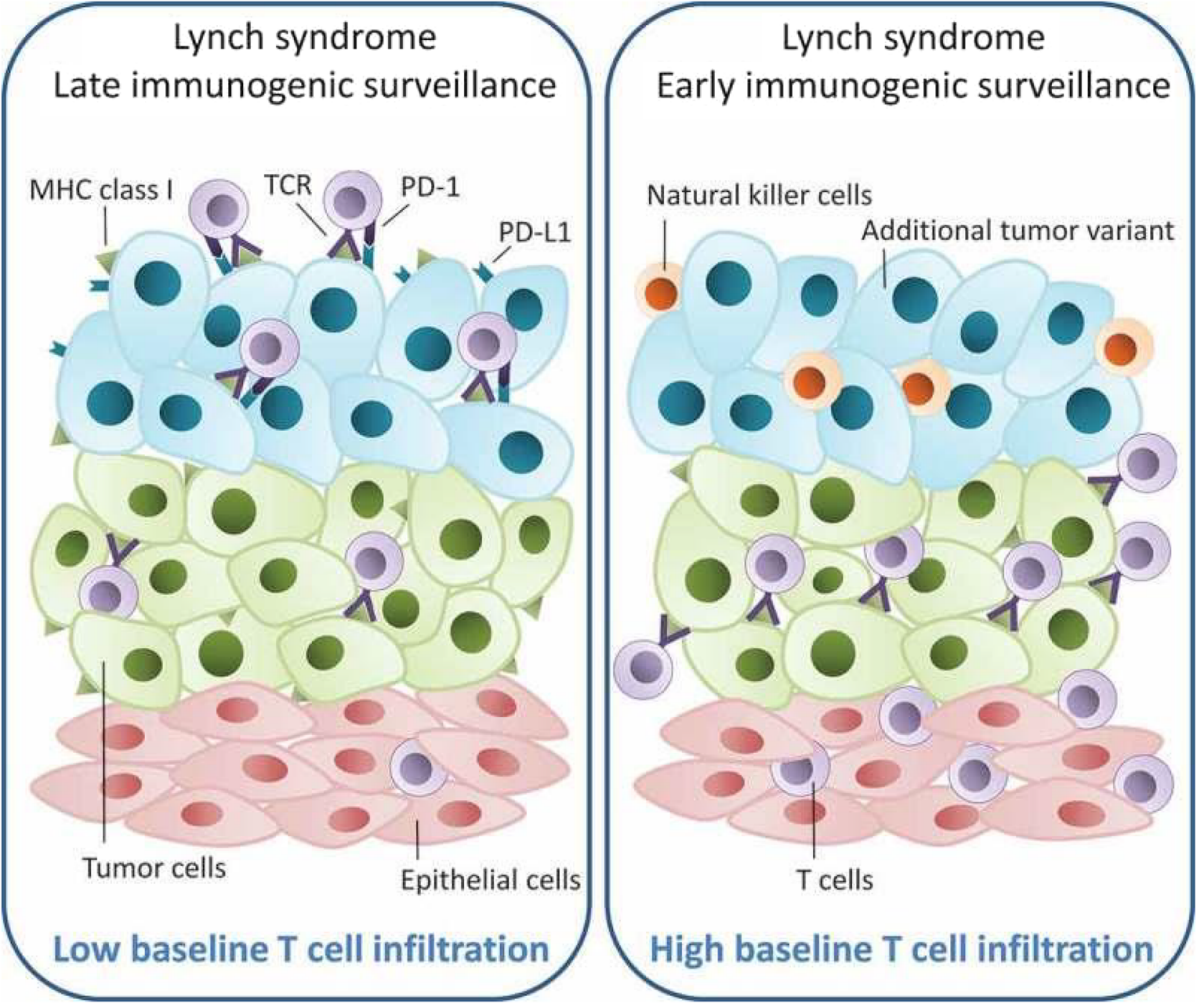
Model showing the possible development of two different immune evasion mechanisms in Lynch syndrome–associated colorectal cancer. When the baseline epithelial tissue is highly infiltrated with CD3 and CD8 T cells (in purple) (right panel), tumor cells (in green) may evade the immune system at early stages, potentially by loss of heterozygosity of B2M and/or MHC class I receptors, which can in turn generate new tumor variants (in blue). Left panel: If the baseline epithelial tissue has a low infiltrative level of T cells, there may be little or no selective pressure on the early tumor cells to avoid the immune system. To avoid immune-mediated killing, tumor cells may upregulate PD-L1, causing T cell exhaustion. Figure from ref. [76]

Several groups have proposed that the processes of early and late immunogenic surveillance in the development of LS-CRC are distinct and thus are influenced and characterized by differing factors (Fig. 6). For example, high baseline CD3^+^, CD8^+^ T cell infiltration may be evident early on, with frameshift mutations in the microsatellite regions of β2M gene, contributing to treatment resistance via loss of β2M, with subsequent loss of MHC class I presentation and decreased sensitivity to cytotoxic T cell–directed therapies (e.g., anti-programmed cell death protein *1* (PD-1) therapies) [37, 76]. On the other end of the spectrum, late immunogenic surveillance is characterized by low baseline CD3^+^, CD8^+^ T cell infiltration, and, in this scenario, there is no selective pressure to evade, but the high mutation rate generates increased neoepitopes (via FSPs), resulting in increased MHC class I presentation, leading to cytotoxic T cell activation and upregulation of PD-L1 inhibitory signals by tumor cells, and thus potentially leading to T cell exhaustion [37, 76]. In essence, there is an ongoing “yin-yang” between (a) frameshift mutations in tumor cells leading to T cell (Fig. 6, purple) recognition of tumor; (b) upregulation of PD-L1 on tumor cells as a consequence of T cell recognition; (c) loss of T cell recognition of peptide-MHC complexes due to β2M mutations; and (d) potential activation of components of the innate immune system (natural killer (NK) cells).

## The Role of Nonsteroidal Anti-Inflammatory Drugs (NSAIDs) in Immune Response and Tumorigenesis in Lynch Syndrome

The use of aspirin in chemoprevention in LS has been explored by investigators of the Colorectal Adenoma/Carcinoma Prevention Programme (CAPP, originally the Concerted Action Polyp Prevention of the European Union). The CAPP2 trial, begun in 1999, randomized 861 individuals known to be LS carriers to 600-mg aspirin or 30-g resistant starch or both in a factorial design. While there was no observed effect at the end of intervention (occurring at an average of 2–5 years), a protective effect of aspirin was demonstrated at an average of 4–6-year follow-up, with a significant reduction in the incidence of LS-CRC, an effect that persisted for greater than a decade. Ultimately, the investigators concluded that taking 600-mg aspirin daily for at least 2 years significantly reduces the risk of CRC cancer in individuals with LS, though the effect may not be apparent for at least 4 years from the commencement of use [86–88]. No evidence was established for short-term risk reduction in adenoma formation or for a protective effect of aspirin on the development of extracolonic cancers in LS carriers [87]. The CAPP3 trial, a dose non-inferiority trial, is currently underway with the first analysis anticipated to occur in 2024. Of interest, the protective effects of aspirin in LS patients has specifically been shown to extend to a subgroup of individuals with obesity as a risk factor for the development of CRC; in LS patients with MLH1 mutation, obesity-related excess CRC risk was abrogated by aspirin use [89].

Other NSAID agents have been suggested to exert a similar effect on LS-CRC risk reduction as aspirin; both ibuprofen and naproxen have been shown to reduce the risk of CRC development in LS patients, with naproxen treatment also contributing to prolonged survival in the same patient population [90, 91]. Vilar and co-investigators provide an in-depth evaluation of the effects of naproxen in both a murine model of LS as well as in patients with LS [91–93]. The apparent risk reduction of LS-CRC may be, in part, attributed to naproxen’s effect on the early stages of tumorigenesis, as a decrease in polyp growth was evident in both LS patients and in mice [91, 94]. In addition to the decrease in intestinal burden in *VpC-Msh2* mice, murine survival was also affected, with increased lifespan demonstrated following treatment with naproxen [94]. Regarding naproxen’s effects on the colorectal mucosa of LS patients, both canonical and non-canonical effects of naproxen may underlie intestinal immune microenvironment modulation in LS (Table 5). In mucosa unaffected by adenoma or tumor, naproxen induced an immune response, with “low”-dose (220 mg) naproxen downregulating genes involved in cell cycle regulation and dynamics at the base of the intestinal crypt (representing the stem cell compartment) and “high”-dose (440 mg) naproxen upregulating immune-related genes while downregulating genes related to cell dynamics at the top of intestinal crypts (constituting the differentiated compartment) [91]. Naproxen also significantly decreased colorectal mucosal levels of PGE2 and other COX-1 and COX-2 metabolites [91]. Naproxen was also shown to activate different immune cell types without increasing lymphoid cellularity [91]. Not only was the activation of different subtypes of T and B cells increased, but an increase in the activation of dendritic cells and macrophages was evident as well (although in the latter two cell populations to a lesser extent). This increase in mucosal immune signals with stable lymphoid cellularity (intraepithelial lymphocytes and mucosa-associated lymphoid tissue) suggests that the lymphocyte expression profile reflects local activation rather than systemic recruitment or lymphoid proliferation, which may reflect enhanced immune surveillance.

**Table 5:** Effects of naproxen on colorectal-associated immune entities in Lynch syndrome patients

• Reduced polyp growth	
• Decreased mucosal PGE2 levels	
• Reduced PGF2, PGD2, thromboxane B2, 9a11b-PGF2a, 6-KetoPGF1 levels	
• Upregulation of immune genes and downregulation of genes related to cell dynamics at top of intestinal crypt (differentiated compartment; “high dose”)	
• Downregulation of genes involved in cell cycle regulation and dynamics at the base of the crypt (stem cell compartment; “low dose”)	
• Increased activation of different subtypes of T and B cells	
• Activation of dendritic cells and macrophages	

Other investigators have also evaluated the effect of naproxen treatment on the same murine model of LS and have observed a reduction of intestinal tumor burden in response to both naproxen and aspirin treatment; further, the introduction of a TGFβRII mutation eliminated the chemopreventive effect of not just one but both NSAIDs without significantly decreasing survival [94]. Tumors that had developed in the presence of TGFβRII mutation also exhibited features reflecting worsening pathologic grade [94].

## MSI and Immunogenicity in LS-CRC

In recent years, much knowledge has been obtained regarding the relationship between the immunogenicity of CRC with dMMR or MSI and systemic treatment response in advanced disease [2–4, 95–105]. The improved clinical outcomes associated with early stage LS-CRCs may be attributed to the combination of multiple factors of which a degree of tumor immunogenicity and an FSP-dependent “auto-vaccination” phenomenon are undoubtedly influential. Unfortunately, these features are not failproof, and some individuals with LS will present with metastatic disease, which is associated with poorer outcomes [106]. Further, although a lower density of immune cell infiltration has been demonstrated in more advanced, lymph node-positive metastatic LS-CRC, the low incidence of metastatic LS-CRC has hindered the ability to fully characterize MSI as a prognostic marker in this setting [72]. Analyses of systemic treatment of MSI-CRC have typically involved patient populations composed largely of individuals with sporadic MSI-CRC, limiting the ability to generate detailed conclusions regarding the small subsets of patients with LS-CRC. With MSI tumors comprising only 3–5% of all stage IV CRCs (and LS-CRCs representing significantly less), a consistent challenge that has plagued prospective studies aiming to evaluate LS-CRC behavior and treatment response is the ability to achieve adequate statistical power secondary to a relatively small population size [107, 108]. Studies examining the benefit of chemotherapy in MSI-CRC have produced contradictory data; moreover, the majority of these studies have also not distinguished between sporadic and inherited MSI-CRCs [102, 109–114]. In 2004, however, a small retrospective study evaluated the survival of LS patients with stage III colon cancers who received adjuvant treatment with 5-fluorouracil and found no significant differences in colon cancer–specific survival or disease-free survival between individuals receiving adjuvant treatment and those who did not undergo treatment with chemotherapy [115]. Treatment options for metastatic LS-CRC, like sporadic MSI-CRC, have remained limited until now.

## Checkpoint Inhibitor MAb Therapy in CRC

Immune checkpoint inhibitor monoclonal antibodies (MAbs) have had a major impact in the treatment of advanced stage MSI-CRC. A fluoropyrimidine/oxaliplatin doublet had remained a first-line standard-of-care for all advanced stage colon cancers for decades, regardless of microsatellite status, until June 2020, when the PD-1 inhibitor pembrolizumab was granted approval by the US Food and Drug Administration (FDA) for the first-line treatment of patients with unresectable or metastatic MSI-H or dMMR CRC [116]. Approval was based on the results of the KEYNOTE-177 trial (NCT02563002), the most recent in a series of studies focused on the effect of pembrolizumab on solid tumors that led to the 2017 landmark FDA approval of the immune checkpoint inhibitor as a tissue-agnostic treatment in patients with unresectable or metastatic solid tumors demonstrated to be MSI-H or dMMR who have progressed after first-line therapy [117–119]. The main efficacy outcome measures in the KEYNOTE-177 trial were progression-free survival (PFS) and overall survival (OS), with median PFS demonstrated to be 16.5 months (95% confidence interval [CI], 5.4, 32.4) in the pembrolizumab arm and 8.2 months in the chemotherapy arm (95% CI, 6.1, 10.2). Overall survival data were not mature at the time of PFS analysis [117]. Nivolumab, another PD-1 inhibitor, was granted accelerated approval in July 2017 for use in patients with metastatic CRC with MSI-H or dMMR who had progressed on a combination of fluoropyrimidine, oxaliplatin, and irinotecan based on the results from the Checkmate-142 trial (NCT02060188), which found an objective response rate (ORR) of 28% (95% CI, 17, 42) in pretreated patients and duration of responses lasting 6 months or greater in 67% (95% CI, 38, 88) of patients [4, 120]. The addition of ipilimumab, an anti-cytotoxic T-lymphocyte antigen 4 (CTLA-4) antibody, to nivolumab as combination therapy in the same cohort of patients further improved ORR to 46% (95% CI, 35, 58) with 89% of responding patients exhibiting durations of response of 6 months or longer, leading to the accelerated approval of the combination regimen in this group of patients in July 2018 [3, 121]. To date, no checkpoint inhibitor MAb has been approved by the FDA for the treatment of MSS-CRC.

## Checkpoint Inhibitor MAb Therapy in LS-CRC

While the option of pembrolizumab as first-line treatment is undeniably groundbreaking for those individuals with MSI-CRC, overall, the question still remains of whether LS-CRC response to this class of agents parallels that of their sporadic MSI-CRC counterparts or is entirely distinct. Considering their immunoreactive differences, increased somatic mutational load, and greater neoantigen burden, LS-CRCs might conceivably exhibit a unique (and perhaps even better) response, but large, prospective, adequately powered trials dedicated specifically to the evaluation of patients with LS-CRC are currently lacking. Several studies assessing the response of individuals with MSI-H or dMMR CRCs to immune checkpoint blockade have found no significant differences between study participants with germline mutation (LS) and those with sporadic MSI-H or dMMR CRC receiving either monotherapy or combination therapy [2–4, 100]. However, although disease control rates were relatively high and responses were reported to be durable, studies were limited by a small subgroup size. To date, there have been no published reports of any large prospective study specifically evaluating the outcomes of LS patients with CRC who have been treated with immune checkpoint inhibitors. Currently, sources of data regarding immune checkpoint inhibitor response in LS-CRC patients remain predominantly limited to case reports and small case series, although one small retrospective study has evaluated the outcomes of LS patients with various LS-associated cancers, including CRC, following treatment with immune checkpoint inhibitors [122–124]. The investigators report that, of the 21 patients identified as having been treated with one of six immune checkpoint inhibitors (ICIs; the majority of whom had received pembrolizumab), 16 had demonstrated either complete response, partial response, or stable disease. Details regarding the specific types of cancers and associated responses are lacking, as well as the MMR status of a substantial fraction of patients, although one of three patients noted to have MSS tumors was described as being responsive to ICI, with continued response at 9 months of therapy [125].

## Neoantigens in Lynch Syndrome as Vaccine Targets

The high degree of immunogenicity associated with LS-CRC and LS-associated extracolonic malignancies may be attributed to the abundance of altered carboxy-terminal peptide sequences generated by coding microsatellite instability-induced shifts of the translational reading frame [48]. These novel FSPs, or neoantigens, are the result of impaired MMR and have been observed to stimulate the adaptive immune system of LS patients with cancer and individuals with LS-associated germline mutations without clinical disease by eliciting antigen-specific T cell responses [44]. In addition to these immune responses, FSP-specific antibodies have also been detected in the sera of LS patients with CRC and LS mutation carriers without cancer [40]. The ability of these neoantigens to evoke such responses in patients with LS-CRC, as well as in individuals with LS who have yet to develop LS-associated cancers, suggests that they may be potential effective targets for vaccine-based therapy. Findings from an open-label single-arm phase I/IIa clinical trial (NCT01461148) evaluating safety and immunogenicity in patients with a history of LS-associated and sporadic dMMR/MSI-CRCs immunized with a MSI-induced FSP neoantigen-based vaccine demonstrated that vaccination was safe, well tolerated, and induced both cellular and humoral immune responses in all vaccinated patients [46]. Further, among these patients who had all been previously treated with at least one modality of standard-of-care treatment, several patients with measurable disease at baseline exhibited stable disease as their best overall response at the time of follow-up [46]. Larger studies are warranted to assess clinical efficacy in both preventive and therapeutic settings.

## Tumor-Associated Antigens as Targets for Vaccine Therapy in Lynch Syndrome

Carcinoembryonic antigen (CEA) and mucin 1 (MUC1) are widely expressed cancer antigens that are well-known to be associated with gastrointestinal malignancies, as well as extracolonic malignancies for which patients with LS are at risk [126–131]. These antigens have been demonstrated to elicit immune responses in individuals with late-stage colorectal adenocarcinomas, as well as in in those with advanced adenomas, making them potential targets for vaccine prevention in individuals who are at high risk for cancer development, such as those with LS [132–136]. Currently, a randomized phase II trial examining the immunogenicity of a MUC1 peptide vaccine with adjuvant in participants diagnosed with advanced adenomatous polyps is ongoing (NCT02134925). While individuals with LS are excluded from participating, the results of this study will hopefully elucidate findings that are relevant and applicable to this patient population, as well. While studies evaluating immunization responses and clinical efficacy in patients with sporadic adenomas and colorectal carcinomas are important to further our knowledge in this area, additional vaccine studies, whether targeting unique mutated neoantigens or widely expressed tumor antigens, are necessary to specifically evaluate responses in patients and family members with Lynch syndrome.

## Potential Vaccine Therapy for LS Polyps

Cancer prevention is the principal objective in the treatment of LS polyps. While risk reduction has largely relied on intense surveillance strategies and procedural intervention, increased knowledge regarding immunosurveillance and immune response in patients with LS has raised the possibility of vaccine-based therapy as a modality of immunoprevention in this setting. Premalignant lesions in LS arise in an active immune microenvironment that is under constant immunosurveillance. This continuous activity has been shown to be present in individuals with LS who have developed tumors, as well as in those who are carriers of germline mutations resulting in dMMR without clinical disease [44]. The factors triggering the shift from a protective effect to an environment favoring immune evasion and, thereby, enabling carcinogenesis in LS are also becoming better understood. An abundance of FSP-induced neoantigens, with their capacity to continuously engage the immune systems of LS patients with and without tumors, and a lifelong predisposition to the development of metachronous and extracolonic malignancies render individuals with LS excellent candidates for vaccine immune intervention. Some potentially promising data have emerged regarding vaccine therapies in CRC; however, these data have been restricted thus far to findings generated from studies of sporadic CRC and adenomas in the general average-risk population [132–136].

Safe and effective immunoprevention is thus a potential strategy for reducing the risk of the development of cancers in Lynch syndrome. Investigators have established a preclinical mouse model in which to develop a cancer preventive vaccine against MSI cancers in LS [137]. Vaccination of LS mice with peptides encoding FSP neoantigens derived from cMS mutations identified in the intestinal tumors of LS mice promoted anti-neoantigen immunity, reduced intestinal tumorigenicity, and prolonged overall survival. In addition, treatment with vaccine in combination with naproxen resulted in even further improved overall survival [137]. Further evidence that the combined use of vaccines and COX2 inhibitors may delay or eliminate LS polyp formation to carcinoma was provided in a study employing a therapeutic CEA-targeting vaccine, designed to induce CEA-specific T cells and celecoxib. CEA transgenic mice were employed in which CEA is a “self-antigen”; these mice were crossed with mice bearing a mutation in the *Apc*^*∆850*^ gene (multiple intestinal neoplasia mice). These progeny mice were shown to spontaneously develop multiple intestinal neoplasms that overexpressed CEA. The vaccine reduced the number of intestinal polyps by 54%, and celecoxib reduced polyps by 65%; the combined use of vaccine plus celecoxib reduced intestinal polyps by 95% and significantly improved overall survival vs either agent alone [138, 139].

## Prospective Studies for the Immunotherapeutic Intervention of LS Polyps

The findings that checkpoint inhibitor MAbs can be employed for clinical benefit of subsets of patients with a range of cancer types and stages have led to an unprecedented level of preclinical and clinical activity involving immune interventions for virtually all cancers. This has coincided with an equally unprecedented level of understanding of the complexities of the immune system and in the interactions between and among different components of both the innate and adaptive immune systems. Studies are revealing that in some cases checkpoint inhibitor MAb alone may induce clinical benefit, while in other cases, additional immune-mediating agents are necessary [140]. Both preclinical and clinical studies are now revealing that to optimize anti-tumor effects one may require (a) an agent to activate T cells directed against a tumor antigen or antigens, (b) potentiating the immune response with the use of agents such as cytokines, (c) reducing or eliminating immunosuppressive entities systemically or in the TME, and (d) altering the phenotype of the premalignant or tumor cells to make them more susceptible to immune-mediated lysis [141]. Agents that have been shown to carry out these various phenomena, either alone or in combination therapies, have all been vetted in preclinical studies and in many cases are in active investigation in clinical studies. The use of many of these immunotherapeutics has now revealed durable clinical responses with, in most cases, acceptable levels of toxicity, especially when compared to many chemotherapeutics and small-molecule targeted therapeutics. Since the cumulative lifetime risk of LS patients developing CRC or other cancers can be as high as 60–80% without surveillance [23], it is timely to consider novel immunotherapeutic approaches for patients suffering with LS polyps.

Since LS polyps have been shown to express known tumor-associated antigens as well as frameshift mutations in the form of potential neoantigens, vaccine therapy approaches must be considered — both as monotherapy or more importantly in combination therapies.

Multiple platforms of therapeutic vaccines designed to initiate tumor antigen–specific CD8+ and CD4+ T cell responses have all been vetted in clinical studies. For example, CEA and MUC-1 are antigens known to present in both CRC and LS polyps. Brachyury is a transcription factor that has been shown to drive the epithelial–mesenchymal transition (EMT) process and has been shown to be expressed in CRC [142–145]. Vaccines targeting CEA, MUC-1, and brachyury alone and in combination have all been shown to generate antigen-specific T cell responses in patients with advanced cancers, with an excellent safety profile and with preliminary evidence of clinical activity [132–134, 146–148]. While vaccines directed against mutated proteins in LS polyps will have a higher degree of specificity than those antigens described above, the level of effort to identify and produce a patient-specific vaccine may well limit its utility.

While vaccine monotherapy is clearly feasible, it is quite possible and more likely probable that benefit for patients with LS polyps will arise from vaccine combination therapies. Four such examples of many potential combinations are given here: (I) *Vaccine plus NSAIDs*: COX2 inhibitors such as celecoxib and naproxen and aspirin have all been shown to have some level of clinical benefit in patients with LS polyps when evaluated by colonoscopy. As mentioned above, the combination of a therapeutic anti-CEA vaccine plus celecoxib was extremely effective in reducing polyps and enhancing survival in a preclinical model compared to either agent alone. (II) *Vaccine plus checkpoint inhibitor MAb*: The use of vaccine alone may initiate a T cell response directed against an antigen expressed in LS polyps, but either inherent checkpoint inhibitor molecules or the induction of checkpoint inhibitor molecules due to the de novo presence of T cells will have the effect of reducing or eliminating the lytic effect of these T cells. In addition to numerous preclinical studies, showing the advantage of vaccine plus checkpoint therapy, this phenomenon has been demonstrated in clinical studies in patients with cervical cancer. A combination of a human papillomavirus (HPV) therapeutic vaccine (shown to induce HPV-specific T cell responses) and a checkpoint inhibitor MAb were able to induce clinical responses in patients with cervical cancer that were not seen with either agent alone [149]. (III) *Vaccine plus cytokine*: As one example, N803 is an IL-15 superagonist immunocytokine. In clinical studies N803 has been shown to enhance both to T cell and NK responses with acceptable toxicity [150]. N803 is currently in multiple phase 2 studies and in a phase 3 study in bladder cancer. Preclinical studies [151] have clearly shown the therapeutic advantage of the use of vaccine in combination with N803, as did preliminary clinical results. Other cytokines or agents to inhibit immunosuppressive cytokines have also been shown to enhance vaccine efficacy in preclinical studies. These include an IL-12 tumor-targeting immunocytokine, an IL-8R inhibitor, and a bifunctional anti-PDL1/TGFβR2 agent [152–155]. (IV) *Vaccine epigenetic modifiers*: Epigenetic modifiers such as the *histone deacetylase* (HDAC) inhibitors entinostat or vorinostat have been shown to alter the phenotype of human tumor cells to render them more susceptible to lysis by T cells and NK cells [156]. Preclinical studies have shown the advantage of the use of HDAC inhibitors with vaccines [157] or other immunotherapeutics in terms of enhanced immune responses and anti-tumor activities.

DNA damage repair inhibitors such as PARPi have shown anti-tumor activity in patients with several tumor types, especially in patients whose tumors are MSI. Clinical responses have been improved with the combined use of PARPi and checkpoint inhibitor MAb [158]. Since many LS polyps are MSI, this combination regimen may also be beneficial in that setting.

The above proposals are examples of the spectrum of immunotherapeutic agents and immune modifiers that can be employed in clinical studies to reduce the clinical burden of LS polyps and ultimate conversion to CRC and/or other neoplasms. As a consequence of the literally hundreds of immunotherapy clinical studies completed, ongoing, and planned in virtually every tumor type, immunotherapy agents are now also becoming the standard-of-care in the neoadjuvant setting and first-line treatment for several cancer types. It is acknowledged that clinical studies in patients with LS polyps are logistically challenging, but the spectrum of immune-mediating agents currently available clearly merits novel clinical studies in patients with this preneoplastic condition.

## Conclusions

Lynch syndrome is the most common form of hereditary colon cancer. Malignant transformation from polyps can occur at accelerated rates, sometimes within 1–3 years, with a high cumulative lifetime risk of developing colorectal cancer and other carcinomas. Few therapeutic options are currently available. There are several immunologic aspects of Lynch syndrome that make it amenable to immune-mediated interventions. These include (a) LS lesions possess microsatellite instability; this in turn leads to the development of a high mutational burden and the development of “novel frameshift peptides” that can act as neoantigens. (b) LS patients develop antibodies and T cell responses to tumor-associated antigens. (c) The tumor microenvironment possesses a T cell infiltrate and a proinflammatory cytokine phenotype. Lynch syndrome lesions, however, also possess immunosuppressive entities such as (a) alterations in MHC class I antigen presentation, (b) TGFβRII mutations, (c) upregulation of PD-L1 on tumor-associated T cells, and (d) tumor-associated FOXP3^+^ regulatory T cells.

The recent renaissance in cancer immunotherapy was spearheaded by the development of checkpoint inhibitor MAbs such as anti-PD1, anti-PDL1, and anti-CTLA4. This has led to literally thousands of clinical studies, which has resulted in durable clinical responses in subsets of patients with a range of cancers including melanoma, bladder cancer, Merkel cell carcinoma, and non-small cell lung cancer, among others. The majority of some tumor types such as CRC and prostate cancer, among others, remain mostly resistant to checkpoint inhibition therapy with the exception of those lesions with microsatellite instability. The 12–15% of CRC lesions that are MSI high (some of which are LS-derived) have a higher response rate to checkpoint inhibition therapy than their MSS counterparts, and the majority of LS polyps are also MSI.

The initial successes in immunotherapy in the treatment of cancer were accompanied with a more detailed understanding of the complexities and dynamics of the human immune system. Consequent preclinical and clinical studies are now demonstrating that to achieve optimal immune-mediated therapies one must understand the mechanisms involved in both host immunity and the TME. A spectrum of immune-mediating agents has recently been developed and interrogated in preclinical studies and ongoing clinical studies that can (a) induce a host response to a tumor-associated antigen, (b) potentiate that immune response, (c) reduce or eliminate immunosuppressive entities in the TME, and (d) alter the phenotype of tumor cells to render them more amenable to immune-mediated attack. To our knowledge, there have been no randomized trials selective for patients with LS polyps or LS-associated carcinomas involving the use of immunotherapeutic agents as monotherapy and, perhaps more importantly, combination immunotherapy regimens. While it is acknowledged that this is logistically challenging, the knowledge gained of the immune properties of LS lesions makes the so-called prevention trials in patients with LS polyps extremely timely and hopefully potentially successful in reducing the development of associated carcinomas.

## Data Availability

Upon request.

## Abbreviations

β2M: Beta-2-microglobulin
CAPP: Concerted Action Polyp Prevention
CEA: Carcinoembryonic antigen
CI: Confidence interval
CRC: Colorectal cancer
CTLA-4: Cytotoxic T-lymphocyte antigen 4
dMMR: Mismatch repair deficient
EMT: Epithelial–mesenchymal transition
FDA: US Food and Drug Administration
FSP: Frameshift peptide
HDAC: *Histone deacetylase*
HNPCC: Hereditary non-polyposis colon cancer
HPV: Human papillomavirus
ICI: Immune checkpoint inhibitor
KIR2DL1: Killer cell immunoglobulin-like receptor 2DL1
LS: Lynch syndrome
LS-CRCs: Lynch syndrome–associated colorectal cancers
MAb: Monoclonal antibody
MLH1: mutL homolog 1
MSH2: mutS homolog 2
MSH6: mutS homolog 6
MMR: Mismatch repair
MSI: Microsatellite instability
MSS: Microsatellite stability
MUC1: Mucin 1
NK: Natural killer
NSAID: Nonsteroidal anti-inflammatory drug
OS: Overall survival
PD-L1: Programmed death-ligand 1
PFS: Progression-free survival
PMS2: Post-meiotic segregation increased 2
TIL: Tumor-infiltrating lymphocyte
TME: Tumor microenvironment
Treg: Regulatory T cell
